# Early Spring, Severe Frost Events, and Drought Induce Rapid Carbon Loss in High Elevation Meadows

**DOI:** 10.1371/journal.pone.0106058

**Published:** 2014-09-10

**Authors:** Chelsea Arnold, Teamrat A. Ghezzehei, Asmeret Asefaw Berhe

**Affiliations:** School of Natural Sciences, University of California Merced, Atwater, California, United States of America; Montana State University, United States of America

## Abstract

By the end of the 20^th^ century, the onset of spring in the Sierra Nevada mountain range of California has been occurring on average three weeks earlier than historic records. Superimposed on this trend is an increase in the presence of highly anomalous “extreme” years, where spring arrives either significantly late or early. The timing of the onset of continuous snowpack coupled to the date at which the snowmelt season is initiated play an important role in the development and sustainability of mountain ecosystems. In this study, we assess the impact of extreme winter precipitation variation on aboveground net primary productivity and soil respiration over three years (2011 to 2013). We found that the duration of snow cover, particularly the timing of the onset of a continuous snowpack and presence of early spring frost events contributed to a dramatic change in ecosystem processes. We found an average 100% increase in soil respiration in 2012 and 2103, compared to 2011, and an average 39% decline in aboveground net primary productivity observed over the same time period. The overall growing season length increased by 57 days in 2012 and 61 days in 2013. These results demonstrate the dependency of these keystone ecosystems on a stable climate and indicate that even small changes in climate can potentially alter their resiliency.

## Introduction

Magnitude and timing of extreme weather events have recently gained attention for their potential to alter ecosystem processes [1]–[4]. The presence of more extreme weather events has increased concerns over the ability of natural ecosystems to respond to such rapid changes [5]. Extreme interannual change in weather (for example, from a very “wet” to a very “dry” year and vice versa) may trigger rapid carbon loss from an ecosystem [6], [7]. For high elevation mountain ecosystems in particular, the seasonal timing of the accumulation and melting of the snowpack is crucial for supplying abundant water to low-lying communities and high-elevation forests [8]. It is also essential for promoting meadow productivity [9] and soil carbon storage [6], [10]. It is expected that earlier snowmelt will result in drying of meadow soils over the course of the growing season. This drying may lead to increased carbon storage through an increase in the net primary productivity of the system, but it can also lead to a loss of carbon through increased rates of decomposition. Whether the ecosystem remains a sink for or shifts to a source of atmospheric carbon dioxide (CO_2_) will have large implications on the ability of the meadow to filter, store, and release water to the river systems. Prolonged conditions that result in a significant loss of carbon can eventually trigger a tipping point to an ecological regime shift in the meadow.

The coupled hydrological and biogeochemical cycles in high elevation meadows are influenced by the depth and duration of the annual winter snowpack that acts as an insulating blanket during the winter [11]. Not only does the winter snowpack protect the meadow soils from large temperature fluctuations and winter desiccation, it also functions to recharge the meadow soils during the spring snowmelt [12]. There is a vital two-way relationship between hydrology and soil organic matter (SOM) dynamics in such high elevation systems. Hydrology exerts a strong control on storage, stability, and composition of SOM [13] in the meadow soils, while SOM dynamics controls the ability of the meadows to provide ecosystem services such as filtering, storing and releasing water to the river systems. Without these wetland systems to slow the passage of water from the snowpack to the streams, the watersheds become less resilient to flood pulses [14]. The essential nature of those ecosystem services warrants a “keystone” status of mountain meadows in terms of mountain hydrology. A keystone species is one that has a disproportionately large impact to an ecosystem in comparison to its abundance. Mountain meadows, though small in aerial extent in the Sierra Nevada, are an essential component of the mountain water cycle. Watersheds that have lost meadow functioning due to degradation have limited water storage capacity and ability to attenuate floods [15]. Degradation of meadows results in a flashy system, where the surface and shallow subsurface flows in the watershed respond rapidly to precipitation events. Furthermore, high-elevation meadows, which are hotspots of biodiversity [16] and function as breeding grounds for many organisms in the central Sierra Nevada Mountains and other similar ecosystems, are likely to be a key indicator of the overall health of the watersheds.

While interannual variations in snowpack depth and duration are normal in the Sierra Nevada [17], consecutive years with extreme water conditions can significantly increase or decrease the overall length of the summer growing season and duration of snow free days, which will directly affect soil carbon storage. Previously, hydrologic modeling research in the Sierra Nevada has highlighted the sensitivity of that region's watersheds to earlier onset of spring and increased duration of low flows [18]. It was shown that some watersheds that are highly vulnerable to an increase in duration of low flows with climate warming, also occupy the largest mountain meadow area. An increase in the duration of low flows can cause meadows systems to dry down significantly, causing feedbacks to ecosystem processes such as primary productivity and soil respiration. If the trend in the onset of spring [19] continues, and meadows dry down earlier in the growing season, we can expect an increase in the decomposition of soil organic matter as the normally saturated soils become aerobic. This could also potentially impact river systems through a reduction in the ability of meadows to contribute to baseflow as they dry down. In addition, the timing of the onset of snowcover in the early winter can impact meadow soils and biota due to the widely fluctuating soil and air temperatures. The meadow soils in theses systems remain at 0°C as soon as snow accumulates in a continuous snowpack. This insulating layer protects overwintering biota and prevents drying of the meadow soil. Colder winter temperatures coupled to lack of continuous snowpack renders meadow soils and biota susceptible to severe desiccation which will impact ecosystem processes such as soil respiration and productivity in the following summer growing season.

An increase in interannual variation in the onset of spring has the potential to dramatically affect the balance between carbon storage and loss. This would occur mainly through changes to the input of carbon from above and belowground biomass [20], and loss through soil respiration and leaching [21], [22] in meadow soils. A period of rapid carbon loss for the organic-rich high-elevation meadow soils can trigger a positive feedback loop that contributes to declining soil moisture [23], further organic matter decomposition and reduced plant productivity through changes in soil structure [24].

In mountain meadow ecosystems, mean changes in the timing of spring snowmelt have already been shown to influence plant phenology [25], [26], interactions between plants and pollinators [27], and longer term changes in meadow vegetation community structure [28]. In order to examine how the timing and duration of snow cover and presence of early season frost events can influence ecosystem processes, (net primary productivity and soil respiration), we monitored changes in surface carbon dioxide flux and above ground net primary productivity over three consecutive summers (2011 to 2013) in two high elevation meadows in the Central Sierra Nevada mountain range of California.

## Methods

### Methodology

The objective of this study was to track ecological responses of high elevation meadows to extreme seasonality. We combine field-based measurements of soil respiration and aboveground net primary productivity with remote sensing techniques to gauge how the amount and timing of precipitation, and seasonality impact meadow systems.

### Site description

**Figure 1:**
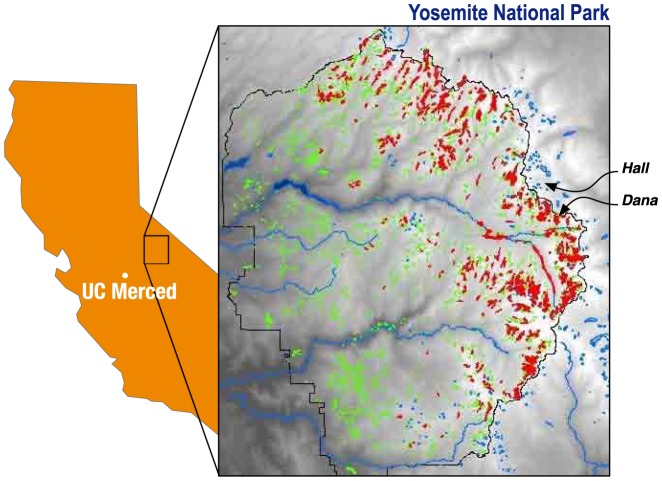
Map of study sites along the boundary of Yosemite National Park, California. Polygons represent the extent of meadow area in Yosemite National Park, with subalpine meadows (>3 hectares and between 2600–3200 m) highlighted in red.

Our study was conducted in two subalpine meadows with different hydrologic regimes located at the crest of the Sierra Nevada mountain range along the boundary of Yosemite National Park (YNP) (Figure 1). Both meadows were formed as a direct result of past glaciation. Their resulting geomorphic position in the landscape remains conducive to high water tables throughout much of the growing season. One meadow is located in the Harvey Monroe Hall Research Natural Area (Hall RNA) at 3200-m elevation on a large medial moraine on the eastern side of the central Sierra Nevada. The mean daily temperatures range from −4.9°C to 12.9°C [29]. The soils in the Hall RNA are characterized as Inceptisols with the suborders Andic Cryumbrepts and Lithic Cryumbrepts [30]. For a contrasting type of meadow, we chose Dana Meadows, which is located at a 3000-m elevation along YNP's Tioga Pass Road in a U-shaped glacial valley with hummocky ablation till. Dana Meadows exhibits mean temperatures similar to those of the Hall RNA and an average precipitation of 1000 mm/year. The soils in Dana Meadows are classified as Inceptisols with the suborders Xeric Dystrocryepts and Vitrandic Eutrocryepts [31]. The research permits for these two study sites were granted by the United States Department of Interior–National Parks, Yosemite National Park for study site Dana Meadows (37.893100N, −119.256900W), and the United States Department of Agriculture–Forest Service, Pacific Southwest Region for study site Hall RNA (37.958056N, −119.296111W). The field studies did not involve endangered or protected species.

### Field methods

In July 2010, transects were established along a hydrologic gradient at two locations in the Hall RNA and two locations in Dana Meadows. The hydrologic gradient in the meadow was established using vegetation associations as a proxy for water table depth [32]. In all four transects, the same vegetation type was utilized to identify each meadow region: *Carex filifolia* in the xeric sites, *Ptilagrostis kingii* in the mesic sites and *Carex scopulorum/Carex subnigricans* in the hydric sites. Three replicate soil collars were inserted in the soil at depths of approximately 3–5 cm (the variation was due to differences in soil characteristics) at three hydrologically distinct regions of the transect (designated as: dry, intermediate, and wet). The collars are located approximately 2 meters apart. The soil carbon dioxide (CO_2_) efflux was measured using a LI-COR 8100A portable infrared gas analyzer (LI-COR Biosciences, Lincoln, Nebraska USA), fitted with a portable 10-cm soil respiration chamber. After a 45-second pre-purge, one-minute measurements were recorded and were followed by a 30-second post-purge. Weekly measurements were recorded during the first half of the growing season, followed by biweekly measurements through September. All measurements were taken at mid-day from collars with vegetation left intact. In each of the 4 transects, there were 6 collars in 2011, and 18 collars in 2012/2013. Above ground productivity was estimated by harvesting the total biomass in six 20 cm square quadrats in each region (dry, intermediate and wet) of the transect at peak production each year. Vegetation samples were oven dried at 50°C, and weighed to determine biomass. Historical and current meteorological data were obtained from the California Department of Water Resources station for Dana Meadows (ID: DAN). Meteorological data utilized for this study include, maximum air temperature, minimum air temperature, snow depth and snow water equivalent (SWE). SWE is the amount of water contained in a unit of snowpack. The April 1 SWE is an important metric for water resource managers in California. It represents the time where historically there has already been the maximum snowpack accumulation for the year, and thus represents the amount of available water to downstream users. Soil temperature and water content were measured at one site in the Hall RNA. Decagon 5TM sensors (Decagon Devices, Inc.) were inserted at 5, 15 and 25 cm below the soil surface. They were continuously monitored using a Decagon EM-50 datalogger.

### Satellite-based remote sensing imagery

The Terra/MODIS surface reflectance (MOD09Q1.5) 8-day L3 global 250-m product was downloaded directly from the Land Processes Distributed Active Archive Center of the United States Geological Survey (https://lpdaac.usgs.gov). This level 3 surface reflectance product, which had been radiometrically corrected and georeferenced, provided a measure of the surface reflectance at the ground level in the absence of atmospheric scattering or absorption. The data were projected in a custom sinusoidal projection specific to the MODIS imagery. The eight-day composite images represented the maximum surface reflectance value for that time period and minimized the impacts of clouds and aerosols.

### Processing MODIS imagery to NDVI

In the first stage of processing, the MODIS product MOD09Q1.5 was reprojected from a custom sinusoidal projection to the California Albers projection. The latter is a version of the Albers Equal Area projection optimized for statewide calculations. Bands 1 (620–670 nm) and 2 (841–876 nm) were utilized to calculate the NDVI over the entire MODIS image. The following equation was used: NDVI  =  (band 2 – band 1)/(band 2 + band 1). The resulting NDVI product was resampled down to 30 m and was used to produce an average NDVI for the entire meadow polygon region. The meadow polygons, resulting derived data layer and the associated metadata are currently being prepared as a spatial data product by the U.S. Geological Survey's Western Ecological Research Center at the Yosemite Field Station [33].

### Statistical Analysis

Soil respiration rates for each collar were integrated over time to determine the cumulative CO_2_ efflux for the growing season. Missing data was filled in via linear interpolation between the prior sampling date and the next date sampled. Repeated measures analysis of variance (RM-ANOVA) was used to determine significant differences between the effects of moisture class across the three years for both ANPP and cumulative CO_2_ flux data. The different sites were utilized as replicates. If the RM-ANOVA model was significant, a Tukey's post hoc test (p<0.05) was used to assess differences between means. In addition, in order to determine the effect of year within a moisture region of the meadow, a subset of data was created for dry, intermediate and wet sites and a one-way RM-ANOVA model was utilized to determine significance within moisture classes across years. If the model was significant, a Tukey's post hoc (p<0.05) was used to determine differences between means. Data was tested for normality prior to analysis using the Shapiro-Wilks test. All statistical analyses were conducted using R statistical software (r-project.org).

## Results and Discussion

### Meteorological Data

**Figure 2:**
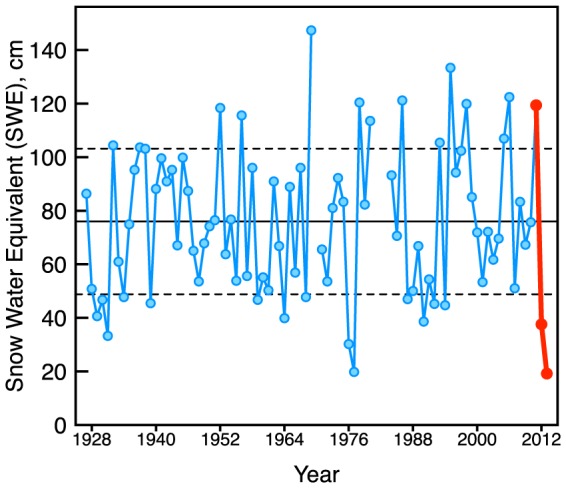
Historic SWE record for Dana meadows (Yosemite National Park) with 2011–2013 highlighted in red.

The last several years in California have been marked by extreme seasonal weather on either ends of the spectrum. The 2011 water year (October 2010 through September 2011) was the seventh-wettest year on record (1929–2012) in YNP, with the April 1 SWE in Dana meadows reaching 156% of the 50 year mean (1951–2000) (Table 1). The 2012 water year was the fifth-driest year on record with only 49% of the mean SWE and the 2013 water year ranked the driest year on record with 25% of the mean SWE (Table 1). Looking at the entire historic record of Dana Meadows SWE, there is an increase after 1969 in the number of years with SWE values greater or less than one standard deviation from the mean (Figure 2). This apparent increase in the SWE variability corresponds to trends found in increase in the variability of streamflow in Central California around the same time period [34].

**Table 1:** Historic ranking of SWE in Dana Meadow.

The top 12 driest years on record (1927–2012)				The top 12 wettest years on record (1927–2012)			
Rank	Driest	SWE (cm)	% of normal	Rank	Wettest	SWE (cm)	% of normal
**1**	**2012**	**19.2**	**25**	1	1969	147.32	192.0
2	1977	19.81	25.8	2	1995	133.35	173.8
3	1976	30.23	39.4	3	2006	122.43	159.6
4	1931	33.27	43.4	4	1986	121.16	157.9
**5**	**2012**	**37.59**	**49.0**	5	1978	120.40	156.9
6	1990	38.61	50.3	6	1998	119.89	156.3
7	1964	39.88	52.0	**7**	**2011**	**119.38**	**155.6**
8	1929	40.64	53.0	8	1952	118.36	154.3
9	1994	44.70	58.3	9	1956	115.57	150.7
10	1992	45.21	58.9	10	1980	113.54	148.0
11	1939	45.47	59.3	11	2005	106.93	139.4
12	1930	46.74	60.9	12	1993	105.41	137.4

Recent years that were in the top 10 wettest or driest years are in bold.

**Figure 3:**
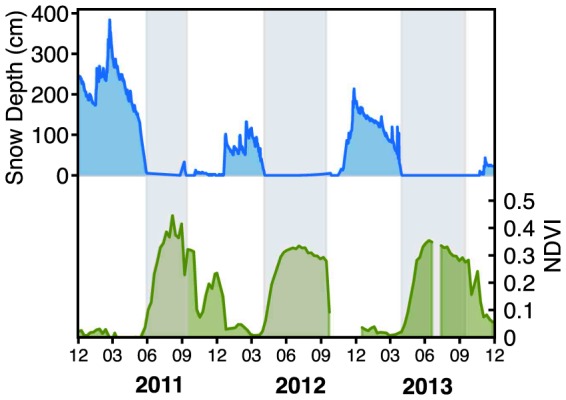
Snow depth (top panel) from Dana Meadows and NDVI (weighted mean average of all subalpine meadows in Yosemite National Park). Gray panels denote the growing season in the meadows as defined by the first day the meadow is snow free and the date where the NDVI crosses a threshold of 0.3.

In ecosystems dependent upon the enduring winter snowpack to insulate them from freezing events, the timing of the first day of continual snow cover for the winter can be critical to biological communities [35]. Likewise, the duration of that snow cover and timing of subsequent spring melt plays an essential role in microbial turnover [36], [37], plant phenology [38], and meadow hydrology [39], [40]. In addition, recent research has shown that winter warming in arctic ecosystems is contributing to a decline in plant productivity during the subsequent summer growing season [41]. Not only was the depth of snowpack distinctly different in the three consecutive years, but also the duration of snow cover differed greatly in all three years (Figure 3). The water year 2012 was especially anomalous with no continual snow cover until mid January. A significant ice storm occurred over the bare soils on December 4, 2012, with widespread needle damage to conifers noted at the study site after snowmelt.

With the exception of January, the average maximum air temperature in Dana Meadows was warmer in 2013 and 2012 through July of each year, as compared to 2011, with the spring (Mar–May) mean temperature increasing by 2–3°C (Table 2). This early warming is a contributing factor to the onset of an early spring in those years. The mean monthly minimum temperatures show warmer spring and summer temperatures in 2012 and 2013 as compared to 2011 (Table 3).

**Table 2:** Mean maximum air temperature (°C) in Dana Meadow.

	2013		2012		2011		2010	Historic Mean (2000–2010)
**JAN**	3.12		4.78		4.93			2.21
**FEB**	3.21	**1.82**	2.82	**3.38**	0.89	**2.53**		2.44
**MAR**	6.81		3.64		2.47			5.35
**APR**	9.11		8.06		5.78			6.62
**MAY**	10.75	**8.89**	11.86	**7.85**	7.67	**5.31**		11.24
**JUN**	15.97		16.28		13.48			15.44
**JUL**	20.05		19.87		18.39			19.62
**AUG**	18.57	**18.20**	21.33	**19.16**	19.48	**17.12**		18.72
**SEPT**	15.11				17.17			14.59
**OCT**	10.43		13.24		11.94			7.06
**NOV**	7.00	**10.85**	6.17	**9.70**	3.68	**10.93**		5.04
**DEC**	4.68		−0.88		2.53		1.76	1.83

Mean seasonal temperatures (DJF, MAM, JJA, SON) are highlighted in gray. Historic mean maximum air temperatures from 2000–2010 are referenced in far right column.

**Table 3:** Mean minimum air temperature (°C) in Dana Meadow.

	2013		2012		2011		2010	Historic Mean (2000–2010)
**JAN**	−11.20		−7.85		−9.48			−10.92
**FEB**	−12.64	**−12.12**	−11.07	**−9.69**	−12.57	**−10.45**		−11.40
**MAR**	−8.01		−9.57		−11.00			−10.11
**APR**	−6.33		−7.18		−9.54			−8.56
**MAY**	−2.03	**−5.46**	−3.39	**−6.71**	−7.35	**−9.30**		−3.75
**JUN**	1.53		0.44		−2.20			0.34
**JUL**	4.87		3.46		2.29			4.25
**AUG**	2.85	**3.08**	5.89	**3.26**	2.76	**0.95**		2.84
**SEPT**	0.17				1.56			−0.93
**OCT**	−4.89		−3.06		−2.62			−4.59
**NOV**	−5.78	**−3.50**	−6.36	**−4.71**	−8.43	**−3.16**		−7.51
**DEC**	−10.09		−12.51		−10.16		−9.28	−10.37

Mean seasonal temperatures ((DJF, MAM, JJA, SON) are highlighted in gray. Historic mean minimum air temperatures from 2000–2010 are referenced in far right column.

### Ecosystem Response

The extreme seasonal changes from 2011 and 2012/2013 caused dramatic shifts in the onset of spring and in the number of snow-free days in the meadows of YNP. In Dana Meadows, the first snow-free day in 2012 and 2013 occurred 57–61 days earlier than in 2011, and the growing season increased by 35–37%, from approximately 106 days in 2011 to 163/167 days in 2012/2013. The documented shift to an earlier onset of spring in the Sierra Nevada [19] appears to have altered the response of the meadow ecosystems; rather than increasing their productivity, the earlier spring has rendered the meadows more sensitive to late winter/early spring frost events [11], [28], [42], [43].

**Figure 4:**
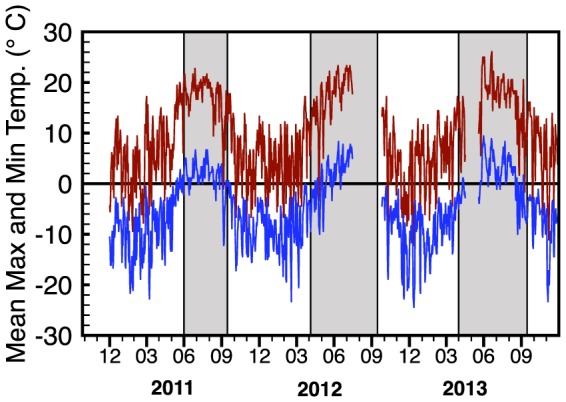
Maximum (red) and minimum (blue) daily air temperatures for Dana Meadow in 2011, 2012 and 2013. Gray panels denote the growing season in the meadow as defined by the first day the meadow is snow free and the date where the NDVI crosses a threshold of 0.3.

**Figure 5:**
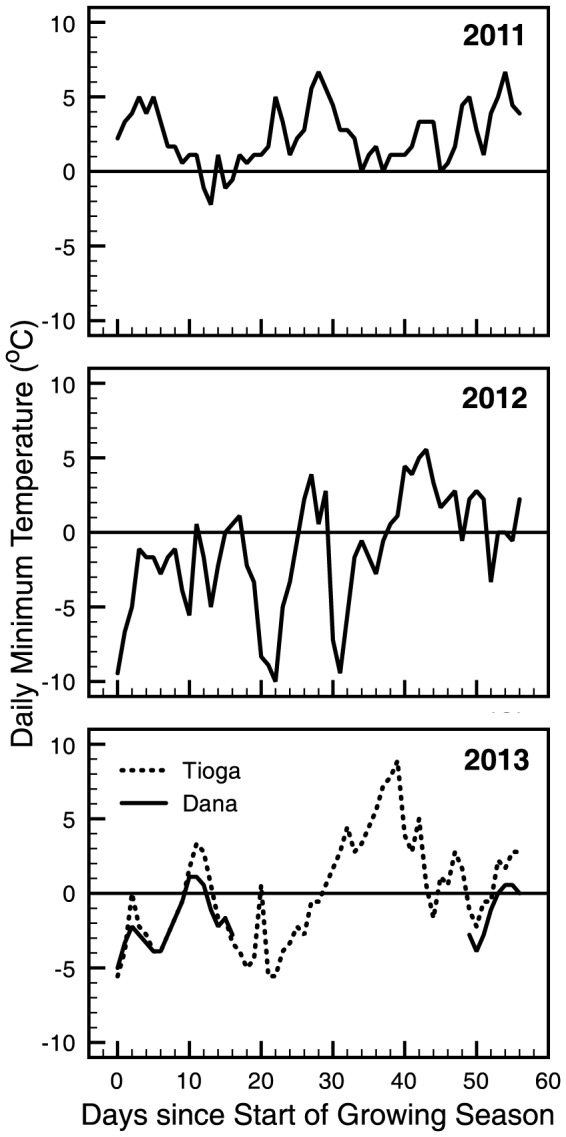
Daily minimum temperatures were used to compare frost events in the first sixty days since the start of the growing season (2011–2013) in Dana Meadow. Growing season was determined by the first day that the meadow was snow free each year. Dotted line represents data from a nearby meteorological station (Station id:TES) at north end of meadow was used for missing data in 2013.

The maximum and minimum temperatures in the meadow show a clear seasonal trend, with very few frost events occurring within a normal growing season (Figure 4). The seasonal trends are similar between years, but the time point when the growing season is initiated is critical for assessing potential frost impacts on newly sprouting vegetation. As the snow melts, it causes saturation of the meadow soil and plants respond rapidly to this moisture and available nutrients by sending up green shoots. This leaves them susceptible to freezing temperatures. Tranquillini (1964) has shown that high elevation plants are very frost resistant, however notes that plants dependent on an insulating snowpack are susceptible to frost damage even in minor frost events [44]. If spring arrives earlier, as in 2012 and 2013, there is an increased likelihood of a severe frost event to damage newly sprouting vegetation. This pattern was evident during two frost events that occurred in 2012 after the snow had cleared from the meadow (Figures 4 and 5). The first event occurred over a four-day period that peaked on May 27, when the temperature dropped to −10°C. This event occurred approximately 20 days into the growing season. The second event occurred over three days beginning on June 5 that included a low temperature of −9.4°C. Because meadows undergo a rapid greening within days of a snowmelt, frost can damage sensitive meadow species and reduce overall productivity [43], [45]. In 2013, there were several frost events that occurred on May 19 and 20 and on May 22 and 23 with a low at −5.5°C.

Frost damage to vegetation was apparent on a larger scale using satellite-derived Normalized Difference Vegetation Index (NDVI) mapping. The NDVI time series for the three years is shown in Figure 3. A rapid greening was apparent at the beginning of the growing season for both years; however, instead of reaching a peak in 2012, the NDVI plateaued before the vegetation senesced in mid-summer, which could indicate that the meadow vegetation was stressed and never reached maximum greenness in 2012. Normal meadow NDVI ranges from 0 during snowcover to a maximum of 0.45–0.65 at peak production and then falls to around 0.3 during senescence. In 2011, the peak NDVI occurred around 0.45, but in 2012 and 2013, the peak NDVI was just above the senescence value. Since the meadow soils begin the season saturated due to snowmelt and dry down over the growing season, it is unlikely that the plateau was caused by the meadows drying earlier in the growing season. If this were the case, we would expect to see a peak NDVI soon after the rapid increase at the beginning of the season. The pattern in 2013 is similar, though there is a small peak in early season NDVI, but the overall pattern is much lower than in 2011, indicating that although aboveground productivity did recover slightly from 2012, the meadow was still in a stressed state. There is an anomalous peak of NDVI that occurs between November 2011 and January 2012. This value was reflective of the senesced vegetation and bare ground that lacked a continuous snowpack until mid January 2012.

**Figure 6:**
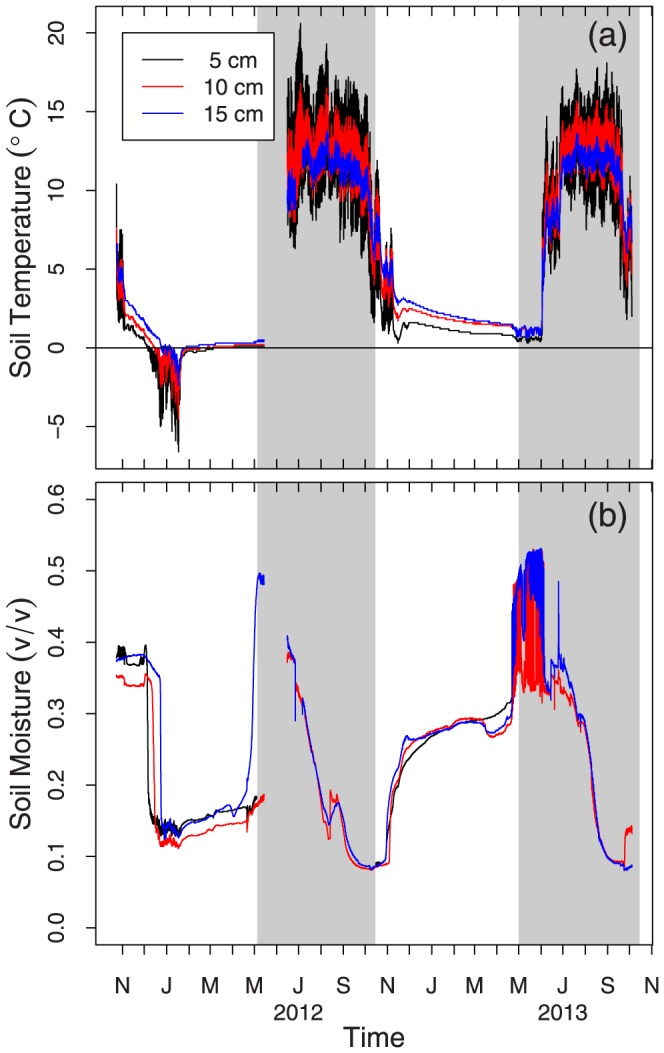
Time series of soil temperatures and volumetric water content for a dry meadow site from November 2011 to November 2013. The shaded panels indicate the growing season in 2012 and 2013.

Another potential explanation for the decreased peak NDVI in 2012 is the winter desiccation of the meadow soils that may have damaged overwintering roots. Figure 6 shows a time series of soil temperature and volumetric water content at three depths in the soil from a drier region of the meadow. The soil temperatures dropped well below freezing for an extended period of time in early 2012 before the snowpack began to accumulate in mid-January. This drop in soil temperature triggered a desiccating event in the soil, as seen in the lower panel. Volumetric soil moisture content was subsequently extremely low before snowpack accumulated. In a similar situation, Bokhorst et al have found that winter warming in the arctic, where snow melts off of the surface renders plants less productive the following spring [46].

**Figure 7:**
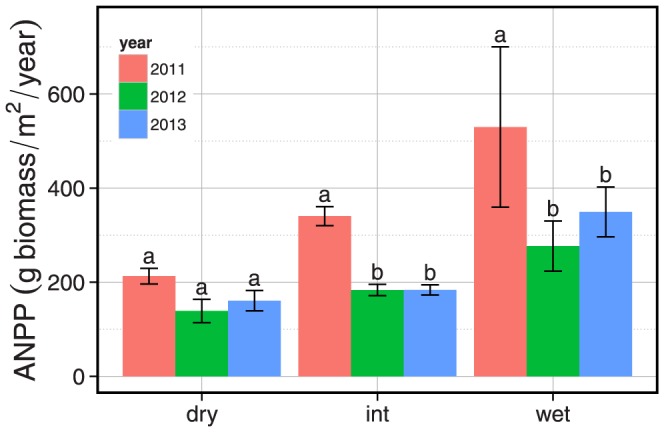
Mean annual aboveground net primary productivity averaged across all four sites for each moisture region in 2011, 2012, and 2013. Error bars represent standard error among sites. Letters denote significant differences in homogenous groups across years as determined by a Tukey post hoc test (p<0.05).

The prolonged growing season in 2012 and 2013 should have led to increased plant productivity if water and temperature were not limiting, but the productivity actually declined by an average of 39% in all the regions of the meadow (Figure 7). Averaged across all sites, the drop in productivity from 2011 to 2012 was the most significant of all three years (p<0.000001). The aboveground productivity in 2013 was still significantly lower than 2011 (p<0.0001) and didn't significantly increase from 2012, indicating that the system is still at a stressed state and not recovering rapidly. However, since belowground biomass was not quantified in this study, it is unknown if the meadow systems are adapting to the change in seasonality by putting more energy into belowground biomass. However, due the fact that meadows already allocate a greater proportion of their carbon inputs (60–80%) to roots, it is unlikely that a shift to more belowground production could offset the carbon losses via soil respiration in 2012 and 2013. Studies have also shown that 90% of the fixed carbon is re-respired in the same season in peats and fens [47]. There is a delicate balance between productivity and carbon loss via respiration and unless the plants drastically increased the belowground biomass in response to the early spring, the losses via soil respiration will override the carbon inputs from the additional belowground biomass.

Different hydrologic regions of the meadows (dry – wet) responded differently to the change in duration and amount of snow cover (Figure 7). There was no significant difference in the productivity of the dry regions of the meadow across the three years. However, both the intermediate and wet regions showed significant reduction in biomass from 2011–2012 (intermediate, p<.0001; wet, p<0.0001). Both regions of the meadow still had significantly lower productivity in 2013 than 2011 (intermediate, p<0.0001; wet, p<0.0001), but no significant change from 2012 to 2013.

**Figure 8:**
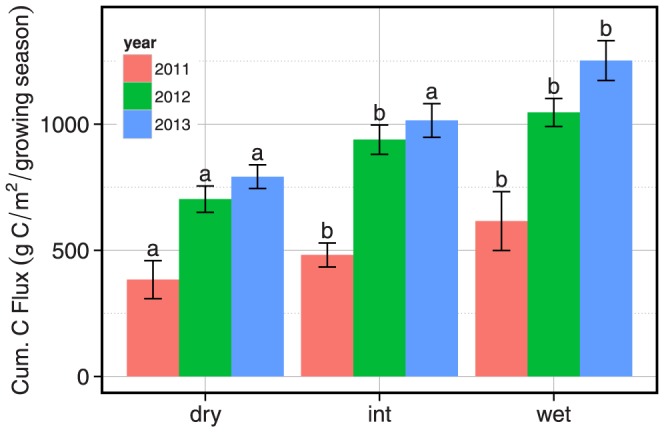
Mean cumulative carbon flux averaged across all four sites for each moisture region in 2011, 2012, and 2013. Error bars represent standard error among sites. Letters denote significant differences in homogenous groups across years as determined by a Tukey post hoc test (p<0.05).

The mean cumulative carbon flux shows an increasing trend over time (Figure 8). Averaged across all moisture regions of the meadows, the mean cumulative carbon flux from the meadows was significant with respect to year (RM ANOVA model; year,p<0.00001, moisture, p<0.00001, moisture:year, p = 0.447). However, this was driven mainly by the significance between 2011–2012 (p<0.00001) and 2011–2013 (p<0.00001). While 2013 continued to have larger cumulative fluxes in all regions of the meadow, they were not significantly different than 2012 (p = 0.481). There was no interaction between moisture region and year. Comparing the individual moisture regions of the meadow, there was significant change in the cumulative carbon flux of the dry, intermediate and wet regions of the meadow from 2011 to 2012 and 2011 to 2013, but no significant change from 2012 to 2013 (Table 4).

**Table 4:** One-Way RM-ANOVA results from homogeneous moisture regions of the meadow.

	Dry	Intermediate	Wet
**2011–2012**	P<0.003	P<0.0007	P<0.006
**2011–2013**	P<0.001	P<0.0004	P<0.002
**2012–2013**	P = 0.798	P = 0.888	P = 0.667

**Figure 9:**
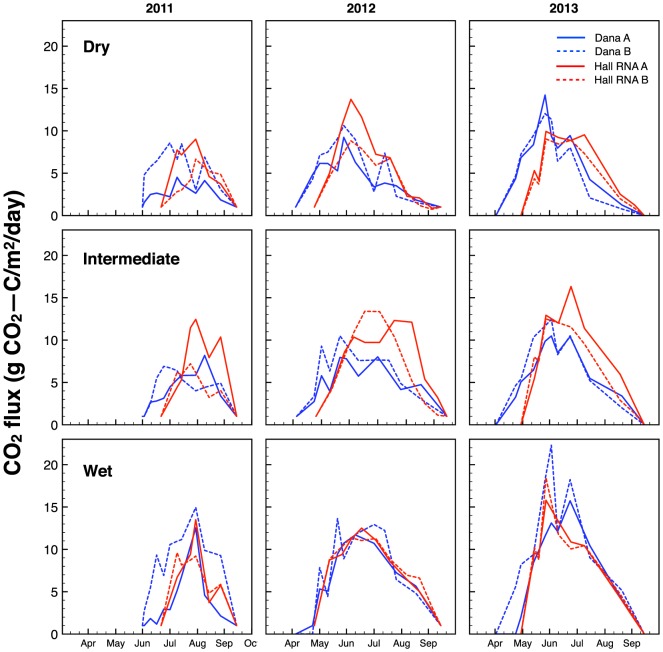
LI-COR surface CO_2_ flux data for 2011, 2012, and 2013 in Dana Meadows and the Hall RNA.

This result suggests that easily decomposable soil organic matter rapidly decomposed with the shift in environmental conditions in 2012 and 2013 [48]–[51]. The short growing season in 2011 and wet conditions throughout the growing season effectively reduced the overall soil carbon efflux in all regions of the meadow. Although the 2011 water year was extremely wet, and the meadows experienced little drying, this had no apparent effect on the available moisture in 2012. One explanation for this finding may be the dryness caused by a lack of snowpack in December 2011 through mid-January 2012; when the snow finally accumulated in January, the meadows were extremely dry underneath the snowpack (Figure 6). These dry soil conditions at the start of the 2012 growing season and below average snowpack coupled to above average surface temperatures throughout the 2012 growing season led to the extreme drying of the meadow soils and subsequently large carbon fluxes. The 2013 water year was the driest on record for Dana Meadows and there was a continuing trend of high mean cumulative carbon flux from the meadows. Although the cumulative flux was similar in both 2012 and 2013, there was a difference in the timing of peak soil carbon efflux. In 2013, the peak soil carbon efflux occurred early in the growing season and rapidly declined in all regions of the meadow, whereas in 2012, the peak occurred in the middle of the growing season (Figure 9). This shift indicates how responsive these ecosystems are to seasonal variation. The sustained magnitude of the cumulative carbon flux from the system over two summers has resulted in a loss of over 6% of the total carbon stock in the meadows we studied.

With climate extremes occurring at an increasing frequency around the world, our data demonstrate that sensitive ecosystems respond rapidly to the changes in seasonality and may reach a tipping point sooner rather than later. Multiple years of ecosystem stresses such as frost or drought can potentially cause a regime shift in vegetation with ramifications to the cycling of carbon in these systems. The magnitude of loss was significant given the small areal extent of these meadows, which is not proportional to their importance to overall ecosystem functioning and keystone position on the landscape. If the frequency of extreme events continues in this region, coupled to a decline in meadow aboveground productivity, we can expect carbon stocks in the meadows to rapidly decline, leading to meadow degradation and a reduction in ecosystem services in these watersheds.

## Acknowledgments

The authors wish to thank J.R. Matchett, Eric L. Berlow, Samuel J. Traina, and John Harte for their comments on earlier versions of this manuscript, and Jesseca Burkhart for help in the field.
